# Phylogenetic niche conservatism and plant diversification in South American subtropical grasslands along multiple climatic dimensions

**DOI:** 10.1590/1678-4685-GMB-2018-0291

**Published:** 2020-04-27

**Authors:** Michel J.F. Barros, Gustavo A. Silva-Arias, Ana Lúcia Anversa Segatto, Maikel Reck-Kortmann, Jeferson N. Fregonezi, José Alexandre F. Diniz-Filho, Loreta B. Freitas

**Affiliations:** 1 Universidade Federal do Rio Grande do Sul, Departamento de Genética, Laboratório de Evolução Molecular, Porto Alegre, RS, Brazil; 2 Universidade Federal de Goiás, Departamento de Ecologia, Goiânia, GO, Brazil

**Keywords:** Altitude, climatic changes, molecular diversification, phylogenetic niche conservatism

## Abstract

Phylogenetic niche conservatism can be investigated at multiple scales on an explicit geographical context. Haplotype-based comparative analyses of lineages occupying the same region, and thus subjected to similar environmental factors, allow decoupling shared evolutionary and ecological patterns, as well as multiple dimensions of adaptive diversification. Here we aimed to assess the role of environmental drivers on diversification of subtropical grassland, based on haplotypic diversity of two plant genera. We sampled two closely related and co-distributed grassland plant genera, *Petunia* and *Calibrachoa,* across their entire distribution area. Eigenvectors extracted from pairwise distances based on chloroplast DNA haplotypes were used to fit Phylogenetic Signal-Representation (PSR) curves to estimate evolutionary patterns in 19 bioclimatic variables and altitude. The PSR curves showed that altitude, precipitation, and temperature variables changed at different rates with haplotype differentiation. Altitude and temperature traits evolved under conditions closer to a neutral dynamics, whereas precipitation traits differentiated following more complex models. Our results indicated that the diversification in the two genera was more limited by precipitation conditions. Based on these novel findings, we suggest that future studies should test the possible impact of precipitation variables on the process of ecological differentiation in these genera.

## Introduction

Niche conservatism has been defined as “the retention of niche-related ecological traits over time”, a definition that is deliberately broad to include different traits and time scales (Wiens *et al*., 2010; see also Peterson, 2011). Within this conceptual framework, Phylogenetic Niche Conservatism (PNC) explains the sharing of ancestral patterns among species as the result of an evolutionary factor that constrains the differentiation of traits and contributes to trait homogenization (Cooper *et al*., 2010; Münkemüller *et al*., 2015). In practice, approaches that test for PNC are useful for evolutionary analysis of a given trait or trait set, usually for species in large clades. Additionally, tests for PNC interrogating a specific underlying model should help validate the many hypotheses and theories found in the literature (Wiens *et al*., 2010; Crisp and Cook, 2012). Results showing a departure from a neutral model can indicate an adaptive bias in trait evolution, although PNC cannot be limited to a signal stronger than Brownian Motion (BM), a reference for random evolution (Cooper *et* al., 2010; Wiens *et al*., 2010).

Another common task for the inference of PNC is to choose among traits, scales and methods of analysis that are currently available for this purpose (Seger *et al*., 2013). Although PNC has been usually analysed at broad phylogenetic scales, it is also interesting to couple such approaches to more phylogeographical and population-level analyses, in a more explicit geographical context (e.g., Diniz-Filho *et al*., 2008). Indeed, the geographical co-occurrence of two or more taxa or lineages is a result of their historical origin and dispersal, as well as common adaptations to past and current climates if species share many ecological and life history traits (Ackerly, 2009; Villalobos *et al*., 2017). Therefore, haplotype-based comparative analyses of taxa or lineages occupying the same region, and thus subjected to similar environmental factors, can expose shared evolutionary and ecological patterns, such as matching habitat evolution. However, comparisons are often made between distantly related taxa and it may be difficult to distinguish the effects of PNC and divergence from those of other evolutionary mechanisms acting over deep time. It is thus important to note that intraspecific haplotype diversity is many times positively correlated to species richness in ecosystems (Papadopoulou *et al*., 2011). As similar evolutionary processes can work at lower phylogeographic or population levels, equivalent haplotype diversity and correlation patterns can be expected. The similarities between genetic diversity and species richness can even reflect common evolutionary patterns and signatures of environmental influences on diversification (Ackerly, 2009).

In fact, the role of the environment on speciation dynamics can now be detected using empirical approaches coupling population divergence at molecular level and ecological divergence patterns (Hua and Wiens, 2013). For instance, in an adaptive radiation process, dramatic rates of molecular diversification underlie the emergence of a number of closely related evolutionary variants, including species in similar habitats within a same area (Condamine *et al*., 2012; Lamont *et al*., 2013; Joly *et al*., 2014). It is also known that fast adaptive divergence towards new environments would be detected through analytical methods that integrate evidence of evolutionary divergence and ecological drivers, while drawing on the distinct ecological characteristics of the niche occupied by each taxon or lineage (Rundle and Nosil, 2005). In this context, the similarity of evolutionary units along different niche dimensions usually exceeds what is expected under a neutral divergence of lineages and may vary among distinct lineages (Cooper *et al*., 2010; Diniz-Filho *et al*., 2010; Gouveia *et al*., 2014).

The extensive grassland areas of southern South America harbour high levels of plant endemism (Fiaschi and Pirani, 2009; Iganci *et al*., 2011). In these regions, grasslands were predominant under cooler and drier glacial weather conditions in both high and lowlands (Behling *et al*., 2004), while during the Pleistocene, savannahs expanded both north and south of the equator, covering large portions of the current grasslands (Behling and Hooghiemstra, 2001; Behling, 2002). Grassland flora is frequently found in periodically cold climates, since a lack of tolerance to the cold limits the presence of other, non-adapted taxa (Donoghue, 2008; Sklenár *et al*., 2011). Consequently, cooler climatic conditions foster the occurrence of many specialized plant species (Bredenkamp *et al*., 2002). To better understand the evolutionary processes underlying this dynamic, it can be helpful to study the effects of historical climate changes on evolution and conservation of taxa facing niche changes, especially when niches are phylogenetically conserved at multiple phylogenetic scales (Wiens *et al*., 2010; Peterson, 2011).

One way to understand the dynamics pointed out above, it is crucial to uncover the relationships among ecological traits and identify those that limit the distribution of taxa, as well as understand the effect of future climatic changes on ecological niches. According Eldon *et al*. (2013), to correctly identify the impact of habitat character istics on the biological processes occurring in complex landscapes, analysed multiple systems should: (i) be widely studied model systems, (ii) have comparable spatial distributions, and (iii) contain several species and/or evolutionary lineages. Following these criteria, we selected the closely related and co-distributed genera *Calibrachoa* and *Petunia* (Solanaceae) to test for patterns in the evolution of niche traits. These genera comprise many lineages and species representative of both lowland and highland grasslands, including several species that are endemic to each of the two types of grassland (at high or low altitude). Both genera have diversified during the Pleistocene (Fregonezi *et al*., 2012, 2013; Reck-Kortmann *et al*., 2014) under the influence of specific, and sometimes similar, ecological forces, which drove the speciation and diversification of the groups inhabiting these connected grassland areas (Fregonezi *et* al., 2013; Barros *et al*., 2015).

The grassland phytoecological units home to *Petunia* and *Calibrachoa* have suffered severe and varied forms of anthropogenic alteration (Behling and Pillar, 2007; Overbeck *et al*., 2007), highlighting the need to study the diversity drivers and environmental forces that have shaped grassland vegetation and allowed the genera persistence. Here, we analyzed samples of all taxa within the genera *Calibrachoa* and *Petunia* using neutral plastid markers (cpDNA) and applied phylogenetic comparative analyses to a haplotypic networks (see Diniz-Filho *et al*., 2012) to evaluate patterns of PNC at population level. This allows us to assess the role of climate features and altitude conditions on diversification at a phylogeographical scale. In this context, our main working hypotheses is that both genera evolved and diversified under similar ecological constraints and that different patterns of diversification could be observed comparing lowland and highland fields.

## Material and Methods

### Characteristics of the phytoecological units and taxa studied

**Figure 1:**
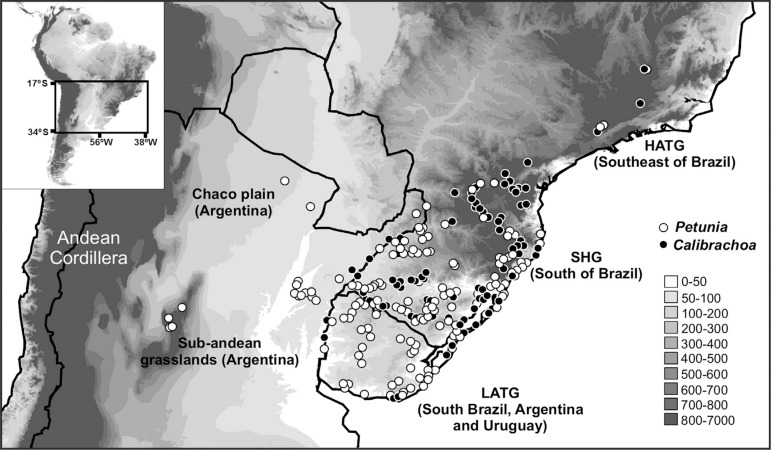
Geographic distribution of the analyzed samples from both genera throughout eastern South America and across altitudinal gradients. Most of the samples were found in Low Altitude Temperate Grasslands (LATG) and Subtropical Highland Grasslands (SHG). Occurrences in High Altitude Tropical Grasslands (HATG) are also indicated, as well as a few records for the Sub-Andean grasslands and Chaco plain vegetation (at left). Shading ranging from white to dark gray represents elevation above the sea level as estimated in meters.

*Calibrachoa* and *Petunia* co-occur in the grasslands of southeastern South America, in an area of varied altitudinal and climatic regimes across Brazil, Uruguay, and Argentina (Figure 1). An initial phylogenetic hypothesis posited an Andean origin for both of these genera as part of the Tribe Petunieae (Olmstead *et al*., 2008) and recent biogeographical reconstructions suggested that, at least in *Petunia* (Reck-Kortmann *et al*., 2014, 2015), the origin lies in the lowland subtropical grasslands of the Pampas, a complex ecoregion dominated by open vegetation of several different types in southern Brazil, Uruguay, and southern Argentina, with a great variety of soils and ecological conditions (Iriondo and Garcia, 1993; Fregonezi *et al*., 2013).

Natural populations of *Calibrachoa* and *Petunia* inhabit three different phytoecological units distinguished by climate and altitude: Low Altitude Temperate Grasslands (LATG) that includes the South Atlantic Coastal Plain (SACP); Subtropical Highland Grasslands (SHG); and High Altitude Tropical Grasslands (HATG). Additionally, some occurrences of these taxa have been reported for the Chaco Plain and the Sub-Andean Grasslands (Figure 1). We chose to focus on the genera *Calibrachoa* and *Petunia* because they are good candidates for the identification of environmental drivers of plant diversification in distinct grassland phytoecological units: several pieces of molecular phylogenetic evidence explain the diversification events in these groups, and for both genera, molecular markers have been mapped (Fregonezi *et al*., 2013). In addition, the availability of new records reduces the possibility of sampling bias.

### DNA isolation, amplification, and sequencing

We chose to analyze maternally inherited cpDNA markers because their sequence diversity reflects largescale patterns of evolution (Oddou-Muratorio *et al*., 2001) and because of their well-known evolutionary models. We used both published DNA sequences (Lorenz-Lemke *et al*., 2010; Fregonezi *et al*., 2013) and recently sequenced ones for all taxa in *Calibrachoa* and *Petunia* (except *P. occidentalis)* in a phylogeographical approach, thus improving the representation of the underlying genetic diversity (Tables S1 and S2).

Total DNA was isolated from dried leaves using a CTAB-based method (Roy *et al*., 1992) and the noncoding intergenic cpDNA regions *trnS-trnG* (Hamilton, 1999) and *trnH-psbA* (Sang *et al*., 1997) were amplified from 16 taxa of *Petunia* and 23 *Calibrachoa* species. The cpDNA markers were amplified and sequenced using previously described protocols (Lorenz-Lemke *et al*., 2006). Amplicons were purified with 20% polyethyleneglycol (Dunn and Blattner, 1987) and sequenced in a MEGABACE 1000 automatic sequencer (GE Healthcare Bio-Sciences Corp., Piscataway, NY, USA) using the DYEnamic ET Terminator Sequencing Premix Kit (GE Healthcare). See Tables S1 and S2 for a complete list of sequences and for details of sources and GenBank accession numbers.

### Identification of genetically-based evolutionary units

In total, we studied 1,821 individuals from 523 geographical locations for *Petunia,* and 568 individuals from 176 locations for *Calibrachoa.* The total sample represents most of the populations of both genera and covers their entire geographic range. We only sampled populations growing in the wild and excluded populations that have recently colonized areas exposed to anthropic disturbance. Sequence analyses were conducted separately for each genus using Chromas 2.0 (Technelysium; Helensvale, Australia). Forward and reverse sequences were aligned separately for each marker and genus using the algorithm Muscle implemented in MEGA7 (Kumar *et al*., 2016), and alignments were manually edited. Variations in poly-A/ T regions of sequences were excluded from the analyses due to the intrinsically high variability and homoplasic conditions of these regions (Lorenz-Lemke *et al*., 2006). Contiguous insertion/deletions (indels) were coded as a single mutational event (Simmons and Ochoterena, 2000). The two marker sequences were then concatenated. Haplotypes were identified using the software DnaSP 5 (Librado and Rozas, 2009). Using one representative sequence per haplotype, we calculated the Tajima-Nei distance (Tajima and Nei, 1984) for all haplotypes in MEGA. In total, we obtained 127 haplotypes comprising 1,174 sites for *Petunia* and 106 haplotypes comprising 1,211 sites for *Calibrachoa* (Table 1).

**Table 1:** Molecular variability of plastid sequences.

Genus	Marker	Length (bp)	V	I	S	Indels
*Calibrachoa*	*trnS-trnG*	765	46	34	55	13
	*trnH-psbA*	446	50	34	46	8
*Petunia*	*trnS-trnG*	713	49	40	49	11
	*trnH-psbA*	461	60	42	63	11

V – variable sites; I – parsimoniously informative sites; S – substitutions; Indels – insertion-deletion events.

### Bioclimatic niches

We obtained 19 bioclimatic variables from the WorldClim 1.4 database (http://www.worldclim.org/bioclim) with a resolution of 2.5 arc-minutes (ca. 4.5 km) to represent the bioclimatic niche of the studied organisms. Altitude values were also included to provide a more general surrogate of the climatic effects. The values of all variables were extracted using the geographical coordinates of all analyzed individuals, and the niche for each haplotype was calculated by averaging the values of all individuals that carried each haplotype. The obtained values can thus be understood as the mean climatic values for the geographical range of each identified haplotype.

### Phylogenetic Signal Representation of bioclimatic variables

Eigenvectors from the genetic distance matrices were obtained by a Principal Coordinate Analysis (PCoA) and used to represented relationships among evolutionary units at multiple hierarchical levels. We used Phylogenetic Eigenvector Regression (PVR) to model the bioclimatic traits by successively adding the eigenvectors and plotting the accumulated coefficients of determination *(R^2^)* against the relative eigenvalues of the accumulated eigenvectors. The resulting Phylogenetic Signal-Representation (PSR) (Diniz-Filho *et al*., 2012) curve provides a description of the evolutionary patterns of the traits under study. For instance, where traits diversify under neutrality (e.g., in a BM process), a linear relationship between *R* ^2^ and eigenvalues is expected. We obtained one PSR curve for each bioclimatic variable, using 999 randomizations and assuming as a null hypothesis the absence of phylogenetic signal in the data.

To allow an easier comparison of evolutionary patterns among the different classes of bioclimatic variables, we also obtained a synthesis of the information contained in the PSR curves by calculating the area contained between each curve and the line expected in a BM scenario, i.e., a line with a slope of 1. The obtained values represent a measure of the deviation from BM. By convention (Diniz-Filho *et al*., 2012), this deviation is considered negative if the curve is below the BM line [e.g., expected under an Ornstein-Uhlenbeck process (O-U)], or positive if it is above the BM line (e.g., diversification burst). The PSR area provides a summary of the shape of the curve and can be used to directly compare different sets of traits (in our case, the bioclimatic niche described by each variable). A trait evolving in an O-U process is usually more easily interpreted as being under evolutionary constraints, such as stabilizing selection, and can be directly associated with PNC. More rarely, it could also be interpreted as a consequence of a very labile trait or convergence (Revell *et al*., 2008).

We analyzed the evolutionary relationships among the identified haplotypes using a median-joining network method (Bandelt *et al*., 2005). In order to explore the relationship between possible bursts in genetic diversification and the bioclimatic variables assessed in this study, we identified the largest changes in the coefficients of determination (R^2^) on the PSR curves *(R^2^* peaks). The eigenvector values linked to such changes were then plotted over haplotype networks (i.e., the eigenvector values for each haplotype were grouped in ranks by evolutionary representativeness using the K-means clustering method).

## Results

### PSR curves of bioclimatic variables

**Figure 2:**
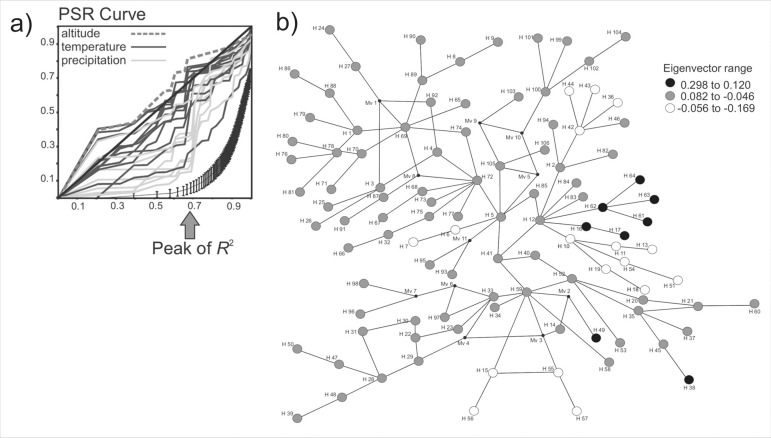
Climatic and altitude variables for *Calibrachoa* and haplotype evolutionary relationships: (a) phylogenetic signal-representation (PSR) curves of altitude (dashed line), temperature (black lines), and precipitation (light gray lines) variables. The lowermost curve with the vertical bars is the null PSR curve, representing the absence of phylogenetic patterns in the data, obtained from 100 randomizations. The diagonal solid line represents the expectation under Brownian Motion evolution. (b) Haplotype connectivity network based on cpDNA intergenic spacers *trnH-psbA* and *trnS-trnG* distance matrices in *Calibrachoa.* Haplotypes are consecutively numbered, with node shades representing the 12^th^PCoA eigenvector, which showed the greatest R^2^ shift (see Table 3 for haplotype details for each species). Eigenvectors choice was based on dramatic changes in the of correlation values, giving origin to the classes that were plotted in the network.

**Figure 3:**
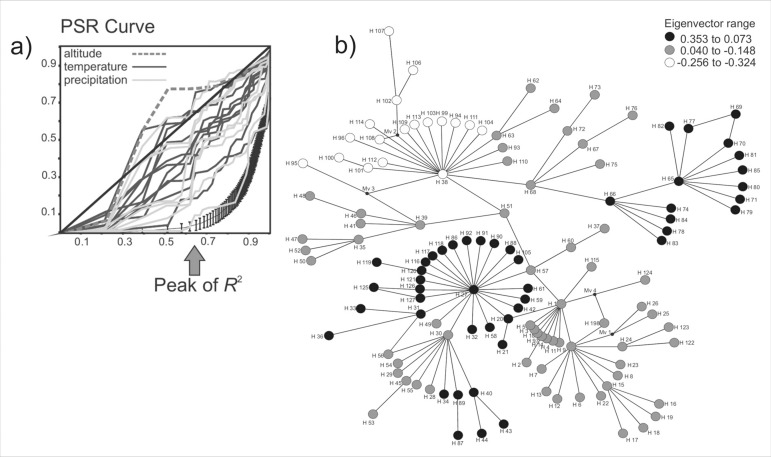
Climatic and altitude variables for *Petunia* andhaplotype evolutionary relationships: (a) phylogenetic signal-representation (PSR) curves of altitude (dashed line), temperature (black lines), and precipitation (light grey lines) variables. The lowermost curve with the vertical bars is the null PSR curve, representingthe absence ofphylogeneticpatterns inthe data, obtainedfrom 103 randomizations. The diagonal solid line represents the expectation under Brownian Motion evolution. (b) Haplotype connectivity network based on cpDNA intergenic spacers *trnH-psbA* and *trnS-trnG* distance matrices in *Petunia.* Haplotypes are consecutively numbered, with node shades representing the 4^th^PCoA eigenvector, which showed the greatest *R^2^* shift (Table 3). Eigenvectors choice was based on dramatic changes in the of correlation values, giving origin to the classes that were plotted in the network.

A total of 80 and 95 eigenvectors were extracted from the haplotype distance matrix, respectively for *Calibrachoa* and *Petunia*. The PSR curves (Table 2) obtained for both genera showed a similar pattern, with major classes of bioclimatic variables (altitude, precipitation, and temperature) showing distinct rates of change with molecular differentiation (Figures 2a and 3a). Temperature and altitude variables tended to have linear PSR representation, whereas precipitation variables were characterized by curvilinear PSRs. The PSR areas for *Calibrachoa* revealed average values for temperature and precipitation variables of -0.02 and -0.14, respectively. The corresponding values for *Petunia* were -0.12 (temperature) and -0.17 (precipitation). The overall values for altitude were 0.08 for *Calibrachoa* and 0.04 for *Petunia,* and both PSR representations were close to linearity.

**Table 2:** Bioclimatic variables used to define altitude (A), temperature (T), and precipitation (P) traits.

Ecological variables	Trait	*Calibrachoa*	*Petunia*
		PSR area	PSR
			area
Altitude	A	0.08	0.04
Annual Mean Temperature	T	0.01	-0.10
Mean Diurnal Range	T	-0.05	-0.16
Isothermality	T	0.09	0
Temperature Seasonality	T	-0.04	-0.07
Max Temperature of Warmest Month	T	-0.01	-0.08
Min Temperature of Coldest Month	T	0.10	-0.11
Temperature Annual Range	T	-0.13	-0.15
Mean Temperature of Wettest Quarter	T	-0.22	-0.32
Mean Temperature of Driest Quarter	T	0.04	-0.10
Mean Temperature of Warmest Quarter	T	0.02	-0.05
Mean Temperature of Coldest Quarter	T	-0.03	-0.17
Annual Precipitation	P	-0.20	-0.13
Precipitation of Wettest Month	P	-0.08	-0.07
Precipitation of Driest Month	P	-0.21	-0.23
Precipitation Seasonality	P	-0.14	-0.39
Precipitation of Wettest Quarter	P	-0.11	-0.07
Precipitation of Driest Quarter	P	-0.25	-0.23
Precipitation of Warmest Quarter	P	0.03	-0.01
Precipitation of Coldest Quarter	P	-0.20	-0.26

**Table 3:** Plastid haplotypes for *Calibrachoa* and *Petunia* species based on combined sequences of intergenic regions *trnS-trnG* and *trnH-psbA.*

Species	Bioregion	Haplotypes
*Calibrachoa* species		
*C. cordifolia* Stehmann & L.W. Aguiar	LATG	H2
*C. excellens* ss*P. excellens* (R.E.Fr.) Wijsman	LATG	H1; H11; H26; H16-H25; H27-H35;
*C. excellens* ss*P. atropurpurea* Stehmann & Semir	LATG	H11; H12; H13; H14; H15
*C. heterophylla* (Sendtn.) Wijsman	LATG	H16; H36-H62
*C. humilis* (R.E.Fr.) Stehmann & Semir	LATG	H63; H64
*C. irgangiana* Stehmann	LATG	H11; H13
*C. linearis* (Hook.) Wijsman	LATG	H65; H66
*C. missionica* Stehmann & Semir	LATG	H84; H85
*C. ovalifolia* (Miers) Stehmann & Semir	LATG	H2; H11; H30, H84H87
*C. pubescens* (Spreng.) Stehmann	LATG	H96
*C. pygmaea* (R.E.Fr.) Wijsman	LATG	H97
*C. sellowiana* (Sendtn.) Wijsman	LATG	H35; H98-H101
*C. thymifolia* (A.St.-Hil.) Stehmann & Semir	LATG	H44
*C. caesia* (Sendtn.) Wijsman	SHG	H1;
*C. dusenii* (R.E.Fr.) Stehmann & Semir	SHG	H3; H4
*C. eglandulata* Stehmann & Semir	SHG	H5;
*C. elegans* (Miers) Stehmann & Semir	SHG	H6; H7
*C. ericifolia* (R.E.Fr.) Wijsman	SHG	H8; H9; H10
*C. linoides* (Sendtn.) Wijsman	SHG	H67-H83
*C. micrantha (R.E.Fr.)* Stehmann & Semir	SHG	H71
*C. paranensis* (Dusen) Wijsman	SHG	H88-H94
*C. sendtneriana* (R.E.Fr.) Stehmann & Semir	SHG	H102-H105
*C. serrulata* (L.B.Sm. & Downs) Stehmann & Semir	SHG	H106
*C. spathulata* (L.B.Sm. & Downs) Stehmann & Semir	SHG	H107-H108
*C. parviflora* (Juss.) D’Arcy	widespread	H95
*Petunia* species		
*P. mantiqueirensis* T. Ando & Hashim	HATG	H1
*P. axillaris* (Lam.) Britton, Sterns & Poggenb	LATG	H31; H32; H33; H34; H36; H38; H40; H41; H42; H44; H45; H46; H47; H48; H50; H51-H62; H65-H67; H69; H70
*P. bajeensis* T. Ando & Hashim	LATG	H76; H77; H78; H120
*P. exserta* Stehmann	LATG	H31; H34; H47; H51; H73;
*P. integrifolia* ss*P. integrifolia* (Hook.) Schinz & Thell	LATG	H31; H34; H46; H79; H80; H81; H82; H83; H84; H86; H87; H89; H90; H91; H92; H104; H114; H121-135; H146; H147; H148
*P. secreta* Stehmann & Semir	LATG	H74; H75
*P. integrifolia* ss*P. depauperata* R. E. Fr	SACP	H46; H79; H80; H81; H82; H85; H89; H93; H96; H97; H99; H100; H101; H102; H103; H115; H116; H117
*P. altiplana* T. Ando & Hashim	SHG	H1; H4-H16
*P. bonjardinensis* T. Ando & Hashim	SHG	H1; H9; H10; H17-H23
*P. reitzii* L. B. Sm. & Downs	SHG	H8; H24
*P. saxicola* L. B. Sm. & Downs	SHG	H8; H24
*P. scheideana* L. B. Sm. & Downs	SHG	H8; H11; H26; H27; H28; H29; H118; H119
*P. inflata* R. E. Fr	SHG/LATG	H31; H104-H110;
*P. interior* T. Ando & Hashim	SHG/LATG	H1; H27; H31; H136-H145

LATG Low Altitude Temperate Grasslands; SHG Subtropical Highland Grasslands; HATGHigh Altitude Tropical Grasslands; SACP South Atlantic Coastal Plain

Peaks in the *R* ^2^ between successive eigenvectors evidenced a possible acceleration in trait diversification (see arrows in Figures 2a and 3a). *Calibrachoa* presented a shared *R* ^2^ peaks pattern among almost all variables, whereas in *Petunia* these peaks were more idiosyncratic.

Although indicators of constrained processes were more commonly observed for precipitation-related variables, one temperature variable – the Mean Temperature of Wettest Quarter – also resulted in small PSR areas for both genera. This variable sits at the intersection of two climatic axes, and although we classified it as a temperature variable, it has a strong precipitation component, strengthening that there was greater constraint to diversification along the precipitation axis for both genera.

### Phylogeographical patterns and niche correlates

The largest R^2^ changes in *Calibrachoa* PSR curves were identified for precipitation variables (Bio13-19) and isothermality (Bio3) between the 12^th^ and 15^th^ eigenvectors. For the *Petunia* PSR curves, the largest R^2^ changes were for precipitation variables (Bio12-19) and isothermality (Bio3) between the 7^th^ and 8^th^ eigenvectors.

The plot of the eigenvectors on the haplotype network in *Calibrachoa* (Figure 2b) represented a peak of successive *R* ^2^ values along the eigenvectors (principally 12^th^ axis) for most of the bioclimatic variables. This appears to represent differentiation of several range-restricted or ecologically specialized species and populations (eigenvector values range 0.298 to 0.120 and -0.056 to -0.169): *Calibrachoa elegans,* isolated at elevations above 1000 m in HATG; *C. excellens* ss*P. athropurpurea,* restricted to the Serra do Sudeste (extreme south of Brazil); *C. heterophylla,* restricted to environments under marine influence in the SACP; and *C. humilis* and *C. linearis,* both restricted to a few LATG localities at the limit between Brazil, Argentina, and Uruguay. The molecular changes underlying the appearance of these species were correlated with ecological traits and indicated phylogenetic signals as a result of more recent phylogeographical lineage expansions in this genus. See Table 3 for haplotypes of each species.

The genus *Petunia* comprised peripheral as well as central haplogroups under the same species groups defined along the peaks in the PSR curves. Those haplogroups were more clearly defined and composed by a greater number of haplotypes. The two subspecies of *P. integrifolia* were included (eigenvector values 0.353 to 0.073 and -0.256 to -0.324; Figure 3b) although some haplotypes belonging to these taxa also clustered with other groups (eigenvector values 0.040 to -0.148; Figure 3b). A small group comprising haplotypes of *P. axillaris* from LATG was observed (eigenvector values 0.353 to 0.073, Figure 3b), deriving from the more central and larger haplogroup that included different haplotypes of *P. axillaris, P. bonjardinensis, P. integrifolia,* and *P. secreta.* These species occur in different areas and present large *(P. axillaris* and *P. integrifolia)* or narrow distribution *(P. bonjardinensis* and *P. secreta).* See Table 3 for haplotype details for each species.

## Discussion

Our findings indicated that bioclimatic variables more related to altitude and temperature evolved mainly under neutrality, close to the Brownian expectation, whereas precipitation-related traits diverged with more curvilinear PSR pattern, similar to expectations under the O-U model. As previously discussed, under PNC, closely related evolutionary units tend to be more similar than expected by chance. Additionally, phylogenetic signals for some traits can serve as a basis to distinguish random patterns from the adaptive peaks generated by phylogenetic signals and conservatism (Losos, 2008; Ackerly, 2009; Diniz-Filho *et al*., 2010). In contrast with divergence or neutrality, PNC can produce a greater uniformity of traits, as well as producing phylogenetic signals (Losos, 2008; Cooper *et al*., 2010). Our comparative phylogeographical analysis of *Calibrachoa* and *Petunia* allowed us to search for phylogenetic signals and PNC by identifying niche dimensions that were subjected to greater evolutionary constraints in both genera: (i) the precipitation was the most conserved trait within the studied phylogenetic groups; (ii) the precipitation variables (Bio13-19) and isothermality (Bio3) may shape phytoecological unit-scale patterns of distribution and diversification in the region; and (iii) the changes observed mostly in temperature traits across geographical space were explained by eigenvectors that correspond to phylogeographical changes along the genera’s geographic range. Therefore, evolutionary studies of molecular diversification under equal or divergent niche conditions should analyse niche variables separately. Temperature gradients had a more linear relationship with phylogeographical patterns deriving from the geographical expansion of these grassland plants. Temperature thus seemed to emerge as a key evolutionary driver. This finding should be further tested in different taxa and biomes. In turn, if precipitation variables constrain phytoecologicalscale patterns and range expansion of haplotypes, as well as regional suitability for molecular diversification, PNC should be common in humidity-related traits, and a new paradigm for phylogenetic and phylogeographical diversification is in order. Considering these two genera, speciation processes and even the phylogenetic relationships should be interpreted on the light of precipitation or precipitation-related variables, instead of complete ecological variable set to represent their niches, as precipitation can dramatically impact these species in these biomes.

If confirmed, these major findings would warrant an update of the theory of niche evolution by emphasizing the partitioning of variables related to temperature, precipitation and altitude in order to allow different associations of traits with different variables of the ecosystem environment, along the diversification pattern expressed by haplotypic variation commonly employed in phylogeographical studies. Indeed, it is interesting to note that phylogeographic lineages are historically related to changes in biome niches (Donoghue, 2008; D’Amen *et al*., 2013) and that there may be a correlation between species and haplotype diversity (Papadopoulou *et al*., 2011). To clarify the evolution of niche traits, it might thus be helpful to describe patterns of haplotype diversification that are common in a biogeographical region and associate these patterns with ecological traits shared among taxa, as performed here. Isolation in space can be caused by many factors and, in highland areas, mainly because the elevation variance (Barros *et al*., 2015). Despite this, the present results showed that precipitation is more directly linked to genetic differentiation within these groups. Finally, the identification of niche properties based on haplotype variability can be crucial to characterize the origin of diversity and may also provide important clues for genetic diversity conservation.

Molecular variability analyses identified several groups under low precipitation seasonality, and these groups were less diverse in areas where precipitation seasonality had higher values. Therefore, areas experiencing lower climatic seasonality and stable precipitation were suitable to species’ maintenance and seem to be diversification hotspots for the grassland taxa studied here. These areas do not necessarily correspond to the differentiation of niche dimensions, but rather represent large-scale suitable areas for these species. Additionally, extreme temperatures have been reported for most of the eastern South American grasslands, with freezing winters and extremely hot summers (Pezza and Ambrizii, 2005; Kottek *et al*., 2006). Grassland vegetation has been subjected to these harsh and extreme conditions for a long time and may therefore be better adapted in its physiology to tolerate seasonal and long-term cyclical temperature changes than changes in precipitation.

The biological properties associated with PNC can cause taxon extinctions in environments undergoing climate changes (Wiens and Graham, 2005). Therefore, detecting patterns of PNC may be helpful to measure the effects of climate changes on diversification, and to identify specific traits underlying local adaptation (Donoghue, 2008; Hua and Wiens, 2013; Oliveira-Filho *et al*., 2013). Geographical expansion and retraction may follow the expansion and retraction of niche variable drivers, which can be reflected in the greater conservation of specific traits at the phytoecological scale.

Based on PSR coefficients, we found that among the analysed bioclimatic variables, seasonality of precipitation presented the stronger influence on genetic structure. Populations inhabiting peripheral areas, such as the HATG, Chaco plain, and Sub-Andean ecosystems, and populations from areas with greater seasonality of precipitation were more divergent than those from core areas (LATG and SHG). These results suggest a lower environmental suitability in peripheral areas, which would represent frontiers of expansion derived from the subtropical areas at the northern limit of the geographical range of both genera (Fregonezi *et al*., 2013). However, the migration to the HATG that lies at the root of the extreme endemism in *Calibrachoa* and *Petunia* does not seem to be a recurrent process, because the connection between the HATG and southern grassland niches is interrupted by tropical forests. Furthermore, even within the SHG, diversification events have given rise to species that are restricted to small pockets of this phytoecological unit (Ando *et al*., 2005; Kulcheski *et al*., 2006; Lorenz-Lemke *et* al., 2010; Fregonezi *et al*., 2013). This suggests the participation of local factors causing endemism. Low molecular divergence was found in populations from regions characterized by low seasonal precipitation (e.g., HATG and peripheral ecosystems), suggesting greater opportunities for dispersal (Crisp and Cook, 2012). However, the mechanism facilitating the dispersal of these barochoric genera (Stehmann *et al*., 2009) across the large high-altitude areas is still unknown.

As discussed above, dispersal would occur during colder climate periods (e.g., at the Last Glacial Maximum, LGM) when grassland phytoecological units expanded and there was greater connection between them (Lorenz-Lemke *et al*., 2010; Fregonezi *et al*., 2013), once peripheral populations show less PNC. Because new environments are colonized by seed dispersal, geographic signals are usually found in uniparental plastid DNA (Oddou-Muratorio *et al*., 2001). These plastid markers are therefore meaningful tools for phylogeographic inference (Beheregaray, 2008; Turchetto-Zolet *et al*., 2013). Nevertheless, pollen-based gene flow and hybridization between areas cannot be discarded, and the examination of other molecular markers can reveal divergent patterns. In addition, if lowland individuals recurrently disperse to the highlands – or vice versa – even at low rates, similarity and admixture would be most likely reflected in nuclear markers. Such patterns would mainly be found among geographical regions that were connected during the LGM. However, community composition is shaped by a combination of historical and contemporary processes, and it is difficult to determine the contribution of each factor in shaping current species ranges (Múrria *et al*., 2017). Variables related to precipitation are associated with a constraint of both genera’s haplotype ranges, possibly reflecting a recent maintenance of species boundaries, related to PNC. The current climate of southern South America is thought to have been established after the Late Holocene period (Behling, 2002), and the diversification of the *Petunia* and *Calibrachoa* genera occurred in different climatic conditions.

In conclusion, precipitation variables constrained the molecular diversification, whereas temperature variables and altitude had a more linear relationship with molecular diversification in both genera, as expected from neutral models. The relationship between niche diversification and phylogeographical range of these genera suggests new evolutionary pathways and improves our understanding of trait adaptation and molecular diversification within and between groups. Our results suggest that different classes of niche traits are best studied separately; in particular, the effects of temperature and precipitation need to be considered independently of one another.

## Supplementary material

The following online material is available for this article:

Table S1 - Sampling information for *Petunia* species.

Table S2 - Sampling information for *Calibrachoa* species.
