# Supplementary material for: Phylogenetic niche conservatism and plant diversification in South American subtropical grasslands along multiple climatic dimensions
Source: Genet Mol Biol. 2020 Apr 27;43(2):e20180291. doi: 10.1590/1678-4685-GMB-2018-0291 (PMC7197982; doi:10.1590/1678-4685-GMB-2018-0291)
Supplement: Table S1 [file 1415-4757-gmb-43-2-e20180291-suppl01.pdf]

Supplementary material to: "Phylogenetic niche conservatism and plant diversification in South American subtropical grasslands along multiple climatic dimensions"

Table S1 - Sampling information for Petunia: average of latitude and longitude per haplotype and GenBank numbers**.**

			Accession Numbers	
**Haplotypes**	**Latitude**	**Longitude**	***trnH-psbA***	***trnS-trnG***
H001	-28.18	-49.89	KF035567	KF035763
H002	-29.05	-50.25	DQ791919	DQ792194
H003	-29.05	-50.25	DQ791923	DQ792198
H004	-28.61	-50.40	DQ791925	DQ792200
H005	-28.61	-50.40	DQ791926	DQ792201
H006	-27.44	-50.00	DQ791927	DQ792202
H007	-28.16	-49.59	DQ791936	DQ792211
H008	-28.43	-50.04	DQ791938	DQ792213
H009	-27.03	-50.11	DQ791939	DQ792214
H010	-28.89	-50.37	DQ791948	DQ792223
H011	-29.06	-50.25	DQ791952	DQ792227
H012	-28.23	-49.72	DQ791991	DQ792266
H013	-28.23	-49.72	DQ791993	DQ792268
H014	-28.60	-49.99	EF660882	EF660873
H015	-28.48	-49.83	DQ791999	DQ792274
H016	-28.40	-49.59	DQ792013	DQ792288
H017	-28.69	-50.29	DQ792024	DQ792299
H018	-28.69	-50.29	DQ792038	DQ792313
H019	-28.69	-50.29	DQ792048	DQ792323
H020	-28.48	-49.89	KJ801271	KJ801287
H021	-28.48	-49.89	KJ801272	KJ801288

			Accession Numbers	
**Haplotypes**	**Latitude**	**Longitude**	***trnH-psbA***	***trnS-trnG***
H022	-27.72	-49.59	DQ792086	DQ792361
H023	-25.23	-50.61	DQ792139	DQ792414
H024	-26.28	-51.80	KF035617	KF035813
H025	-25.51	-51.62	DQ792171	DQ792446
H026	-25.51	-51.62	DQ792172	DQ792447
H027	-30.63	-53.93	DQ225613	DQ225371
H028	-29.93	-55.91	DQ225617	DQ225375
H029	-30.68	-55.85	DQ225662	DQ225420
H030	-31.24	-55.40	JF917467	JF918024
H031	-34.53	-55.02	DQ225609	DQ225367
H032	-34.78	-54.68	DQ225617	DQ225375
H033	-34.40	-53.78	DQ225619	DQ225377
H034	-34.40	-53.78	JF917533	JF918090
H035	-31.45	-55.98	DQ225621	DQ225379
H036	-33.88	-54.62	JF917565	JF918122
H037	-32.94	-54.31	JF917576	JF918133
H038	-30.55	-54.25	JF917582	JF918139
H039	-31.27	-55.51	JF917585	JF918142
H040	-31.25	-54.32	JF917590	JF918147
H041	-31.20	-55.78	JF917644	JF918201
H042	-30.87	-53.45	JF917396	JF917953
H043	-30.57	-53.39	DQ225611	DQ225369
H044	-30.22	-52.57	JF917415	JF917971
H045	-32.14	-56.11	JF917698	JF918255
H046	-31.81	-56.22	JF917704	JF918261
H047	-31.81	-56.22	JF917419	JF917975
H048	-31.14	-55.91	JF917714	JF918271
H049	-30.44	-57.98	JF917723	JF918280

H050	-30.57	-56.60	JF917724	JF918281
H051	-30.55	-58.01	JF917727	JF918284

			Accession Numbers	
**Haplotypes**	**Latitude**	**Longitude**	***trnH-psbA***	***trnS-trnG***
H052	-31.31	-57.09	JF917740	JF918297
H053	-31.33	-57.33	JF917423	JF917979
H054	-30.01	-56.22	JF917778	JF918336
H055	-29.56	-57.51	JF917792	JF918350
H056	-30.20	-58.79	JF917439	JF917996
H057	-28.22	-62.53	JF917854	JF918412
H058	-30.86	-64.51	JF917453	JF918010
H059	-30.88	-53.42	AY772892	DQ225424
H060	-30.55	-53.55	AY772897	KC832915
H061	-30.55	-53.55	KJ801273	KJ801289
H062	-31.41	-54.13	KF035436	KF035629
H063	-31.01	-54.43	KJ801282	KJ801298
H064	-30.96	-54.65	KJ801283	KJ801299
H065	-31.37	-51.32	DQ208162	DQ208028
H066	-29.11	-49.50	DQ208122	DQ207991
H067	-29.88	-50.39	DQ208132	DQ208001
H068	-29.82	-50.18	GQ455638	GQ477730
H069	-30.94	-50.74	DQ208137	DQ208006
H070	-30.94	-50.74	DQ208139	DQ208008
H071	-32.52	-52.50	KF035463	KF035656
H072	-30.08	-51.12	KJ604194	KJ604448
H073	-30.08	-51.12	KJ604195	KJ604449
H074	-28.99	-49.40	DQ208125	DQ207994
H075	-29.86	-50.06	KJ604211	KJ604465
H076	-29.91	-50.43	KJ604219	KJ604473
H077	-30.94	-50.74	DQ208142	DQ208011

H078	-29.87	-50.07	KJ604251	KJ604505
H079	-31.45	-51.22	KJ604319	KJ604573

			**Accession Numbers**	
**Haplotypes**	**Latitude**	**Longitude**	***trnH-psbA***	***trnS-trnG***
H080	-32.00	-52.22	KJ604337	KJ604591
H081	-32.52	-52.50	KJ604346	KJ604346
H082	-32.60	-52.49	KJ604350	KJ604604
H083	-28.84	-49.43	KJ604413	KJ604667
H084	-28.84	-49.43	KJ604414	KJ604668
H085	-33.91	-53.51	KJ604431	KJ604685
H086	-27.25	-53.94	DQ208148	DQ208017
H087	-28.30	-54.26	DQ208150	DQ208019
H088	-28.46	-55.13	KF035489	KF035682
H089	-28.23	-55.61	KF035490	KF035683
H090	-27.88	-55.06	KF035500	KF035693
H091	-27.84	-54.63	KF035504	KF035697
H092	-27.84	-54.63	KF035505	KF035698
H093	-27.41	-53.91	KF280771	KF280827
H094	-30.66	-51.39	KJ024582	KJ024577
H095	-32.13	-52.17	KJ604340	KJ604594
H096	-30.41	-51.20	KF035484	KF035677
H097	-26.75	-51.04	KF280776	KF280832
H098	-26.75	-51.06	KF280784	KF280840
H099	-30.97	-54.27	KM982260	KM982344
H100	-30.87	-55.09	DQ208110	DQ207980
H101	-30.81	-55.59	KF035511	KF035704
H102	-29.89	-55.02	DQ208118	DQ207988
H103	-30.23	-51.33	KF035476	KF035669
H104	-30.23	-51.33	KF035477	KF035670
H105	-29.55	-53.79	KF035551	KF035744

H106	-32.69	-58.20	KJ801281	KJ801297
H107	-30.57	-54.44	KF280764	KF280820

			**Accession Numbers**	
**Haplotypes**	**Latitude**	**Longitude**	***trnH-psbA***	***trnS-trnG***
H108	-30.48	-53.16	DQ208094	DQ207964
H109	-31.09	-53.80	DQ208096	DQ207966
H110	-30.55	-53.55	DQ208098	DQ207968
H111	-30.55	-53.55	GQ455474	GQ477566
H112	-30.29	-52.82	KJ801281	KJ801297
H113	-30.22	-52.57	DQ208103	DQ207973
H114	-30.22	-52.57	KF035560	KF035756
H115	-28.88	-52.43	KF035572	KF035768
H116	-28.05	-53.69	KF035577	KF035773
H117	-28.38	-54.04	KF035592	KF035788
H118	-28.35	-54.28	KF035595	KF035791
H119	-27.77	-53.82	KF035607	KF035803
H120	-27.63	-53.56	KF035611	KF035807
H121	-27.63	-53.56	KF035614	KF035810
H122	-27.09	-52.79	KF035620	KF035816
H123	-26.28	-56.22	KJ801279	KJ801294
H124	-26.63	-54.10	KJ801278	KJ801295
H125	-27.26	-53.98	KF280789	KF280845
H126	-27.26	-53.98	KF280792	KF280848
H127	-27.26	-53.98	KF280796	KF280852
