# Supplementary material for: Phylogenetic niche conservatism and plant diversification in South American subtropical grasslands along multiple climatic dimensions
Source: Genet Mol Biol. 2020 Apr 27;43(2):e20180291. doi: 10.1590/1678-4685-GMB-2018-0291 (PMC7197982; doi:10.1590/1678-4685-GMB-2018-0291)
Supplement: Table S2 [file 1415-4757-gmb-43-2-e20180291-suppl02.pdf]

Supplementary material to: "Phylogenetic niche conservatism and plant diversification in South American subtropical grasslands along multiple climatic dimensions"

Table S2. Sampling information for *Calibrachoa*: Average of latitude and longitude per haplotype and GenBank numbers.

			Accession Numbers	
**Haplotypes**	**Latitude**	**Longitude**	***trnH-psbA***	***trnS-trnG***
H01	-25.90	-49.48	JX178636	JX178636
H02	-29.42	-53.72	JX178637	JN565824
H03	-25.48	-49.79	JX178638	JN565825
H04	-25.48	-49.79	KM982181	KM982261
H05	-27.40	-49.50	JX178639	JN565826
H06	-20.48	-44.01	JX178640	JN565827
H07	-20.09	-43.98	KM982182	KM982262
H08	-24.98	-50.10	KM982183	KM982263
H09	-24.77	-50.15	KM982184	KM982264
H010	-30.48	-53.25	KM982185	KM982265
H011	-30.54	-53.55	KM982186	KM982266
H012	-31.12	-54.59	KM982187	KM982267
H013	-30.54	-53.56	KM982188	KM982268
H014	-30.32	-51.02	KM982189	KM982269
H015	-29.82	-54.16	KM982190	KM982270
H016	-30.59	-53.52	KM982191	KM982271
H017	-30.59	-53.52	KM982192	KM982272
H018	-30.59	-53.52	KM982193	KM982273
H019	-30.59	-53.52	KM982194	KM982274
H020	-28.20	-48.69	KM982195	KM982275

H021	-28.20	-48.69	KM982196	KM982276
H022	-28.15	-49.72	KM982197	KM982277

			**Accession Numbers**	
**Haplotypes**	**Latitude**	**Longitude**	***trnH-psbA***	***trnS-trnG***
H023	-28.15	-49.72	KM982198	KM982278
H024	-25.48	-51.54	KM982199	KM982279
H025	-25.48	-51.54	KM982200	KM982280
H026	-25.74	-51.62	KM982201	KM982281
H027	-25.84	-51.62	KM982202	KM982282
H028	-29.92	-51.60	KM982203	KM982283
H029	-30.04	-51.12	KM982204	KM982284
H030	-30.05	-51.12	KM982205	KM982285
H031	-30.05	-51.12	KM982206	KM982286
H032	-25.67	-50.63	KM982207	KM982287
H033	-27.46	-50.87	KM982208	KM982288
H034	-30.92	-51.50	JQ072006	JQ082455
H035	-29.66	-50.11	JQ072007	JQ082456
H036	-30.98	-51.17	JQ072010	KM982289
H037	-29.43	-49.80	KM982209	JQ082457
H038	-29.66	-50.11	JQ072011	KM982209
H039	-29.43	-49.80	KM982210	KM982291
H040	-30.54	-50.42	KM982291	KM982291
H041	-30.94	-50.74	JQ072009	KM982293
H042	-31.83	-51.72	KM982212	JQ082459
H043	-31.11	-50.90	KM982213	JQ082460
H044	-32.10	-51.99	KM982214	JQ082463
H045	-29.89	-50.43	KM982215	JQ082462
H046	-31.67	-51.43	JQ072012	KM982215
H047	-32.99	-52.73	JQ072014	KM982295
H048	-32.99	-52.73	KM982216	JQ082466

Haplotypes	Latitude	Longitude	Accession Numbers	
			trnH-psbA	*trnS-trnG*
H049	-32.99	-52.73	KM982217	KM982296
H050	-32.99	-52.73	JQ072015	KM982217
H051	-31.73	-51.94	JQ072016	JQ082467
H052	-30.44	-51.19	KM982218	KM982298
H053	-30.41	-51.20	JQ072017	KM982299
H054	-30.41	-51.20	KM982219	JQ082468
H055	-29.89	-54.85	JQ072018	KM982300
H056	-29.89	-54.85	JQ072019	KM982301
H057	-29.89	-54.85	JQ072020	KM982302
H058	-29.85	-54.91	JQ072021	JQ082469
H059	-29.85	-54.91	KM982220	KM982303
H060	-28.46	-48.77	JQ072022	JQ082470
H061	-29.42	-56.69	JX178645	KM982304
H062	-28.88	-56.53	KM982221	KM982305
H063	-31.40	-58.08	JX178647	N565833
H064	-32.18	-58.17	KM982222	KM982306
H065	-26.21	-49.31	KM982223	KM982307
H066	-26.25	-49.46	KM982224	KM982308
H067	-27.31	-50.39	KM982225	KM982309
H068	-26.74	-49.82	KM982226	KM982310
H069	-26.04	-49.54	KM982227	KM982311
H070	-26.90	-49.73	KM982228	KM982312
H071	-27.56	-50.01	KM982229	KM982313
H072	-27.19	-50.38	KM982230	KM982314
H073	-27.56	-50.01	KM982231	KM982315
H074	-27.56	-50.01	KM982232	KM982316
H075	-26.74	-49.42	KM982233	KM982317
H076	-28.04	-49.61	KM982234	KM982318

			Accession Numbers	
**Haplotypes**	**Latitude**	**Longitude**	***trnH-psbA***	***trnS-trnG***
H077	-26.51	-51.17	KM982235	KM982319
H078	-27.56	-50.01	KM982236	KM982320
H079	-24.43	-47.37	KM982237	KM982321
H080	-27.56	-50.01	KM982238	KM982322
H081	-27.55	-49.98	KM982239	KM982323
H082	-27.75	-54.84	KM982240	KM982324
H083	-27.80	-55.30	KM982241	KM982325
H084	-30.81	-55.62	KM982242	KM982326
H085	-30.46	-56.28	KM982243	KM982327
H086	-25.46	-49.65	KM982244	KM982328
H087	-25.46	-49.65	KM982245	KM982329
H088	-25.46	-49.65	KM982246	KM982330
H089	-25.47	-49.66	KM982247	KM982331
H090	-25.47	-49.66	KM982248	KM982332
H091	-25.47	-49.66	KM982249	KM982333
H092	-25.47	-49.72	KM982250	KM982334
H093	-30.63	-52.91	JX178654	DQ208029
H094	-28.37	-54.88	JX178655	JN565839
H095	-30.35	-56.28	KM982251	KM982335
H096	-28.21	-49.79	KM982252	KM982336
H097	-28.36	-49.60	KM982253	KM982337
H098	-28.21	-49.79	KM982254	KM982338
H099	-28.39	-49.54	KM982255	KM982339
H100	-28.41	-49.56	KM982256	KM982340
H101	-28.35	-49.57	KM982257	KM982341
H102	-28.35	-49.56	KM982258	KM982342
H103	-28.38	-49.56	JX178657	JN565842
H104	-28.39	-49.54	JQ082473	JN565843
H105	-26.43	-51.24	KM982259	KM982343

H106	-26.29	-51.49	JX178659	JN565846
